# Revised French guidelines for the diagnosis and management of migraine in adults and children

**DOI:** 10.1186/1129-2377-15-2

**Published:** 2014-01-08

**Authors:** Michel Lanteri-Minet, Dominique Valade, Gilles Geraud, Christian Lucas, Anne Donnet

**Affiliations:** 1 Departement d’Evaluation et de Traitement de la Douleur, Hopital Cimiez, 4, avenue Reine-Victoria, 06000 Nice, France; 2 INSERM/UdA, U1107, Neuro-Dol, 8, place Henri-Dunant, BP38, 63001 Clermont-Ferrand, France; 3 Centre Urgence Cephalees, Hopital Lariboisiere, 2, rue Ambroise-Pare, 75010 Paris, France; 4 Service de Neurologie, Hopital Rangueil, 170, avenue de Casselardit, Toulouse 31300, France; 5 Service de Neurologie et Pathologie Neurovasculaire, Hopital Salengro, rue Emile-Laine, Lille 59037, France; 6 Centre d’Evaluation et de Traitement de la Douleur, Hopital Timone, 264, rue Saint-Pierre, Marseille cedex 05 13385, France

## Background

### Sponsor

These revised guidelines were prepared at the request of the *Société Française d'Etude des Migraines et des Céphalées* (SFEMC; French Society for the Study of Migraine Headache). They are a revision of the professional guidance on the “Diagnosis and therapeutic management of migraine in adults and children: clinical and economic aspects” published by the ANAES in 2002 and revised in 2012.Scope of the guidlines.

These guidelines concern the overall management of migraine, diagnostic and therapeutic strategies, economic aspects of the disease and its treatments, menstrual (catamenial) migraine, migraine in pregnancy, migraine and oral contraception, migraine and the menopause.

Headaches other than migraine will not be discussed except in the context of differential diagnosis. Other subjects not discussed in these guidelines include diseases associated with migraine apart from psychiatric problems, risk factors, migraine and smoking, chronic migraine rare forms and complications of migraine.

### Patients concerned by the guidelines

These guidelines concern adults and children.

### Professionals concerned by the guidelines

These guidelines are aimed at all professionals involved in the management of patients with migraine, including general practitioners (GPs), specialists and retail pharmacists.

### Grade of recommendations and study methodology

The recommendations proposed have been classed as grade A, B or C as follows:

(i) a grade A recommendation is based on scientific proof established by studies with a high level of evidence such as adequately-powered comparative, randomised trials without major bias, or comparative, randomised meta-analyses or decision analyses based on well-conducted studies.

(ii) a grade B recommendation is based on a scientific presumption provided by studies with an intermediate level of proof, such as randomised, comparative trials with low power, cohort studies, well-conducted non-randomised comparative studies or cohort studies.

(iii) a grade C recommendation is based on studies with a lower level of proof such as case–control studies or case series.

In the absence of proof, the recommendations proposed are based on professional agreement between members of the working group. The absence of a level of proof does not signify that the recommendations are not pertinent and useful. The absence of proof should prompt complementary studies wherever possible.

Revision of these recommendations was carried out by the SFEMC, while respecting AGREE methodology. The working group was divided into four sub-committees, each attributed a particular set of themes, a coordinator and a number of participants:

(i) diagnosis and complementary examinations: coordinator: Gilles Géraud (neurologist); participants: Pierric Giraud (neurologist), Evelyne Guegan-Massardier (neurologist)

(ii) handicap-epidemiology-socioeconomic cost: coordinator: Dominique Valade (neurologist); participants: Geneviève Demarquay (neurologist), André Pradalier (internal medicine)

(iii) acute treatment of migraine: coordinator: Christian Lucas (neurologist); participants: Gilles Baudesson (GP), Anne Ducros (neurologist), Serge Iglesias (neurologist), Claire Lejeune (internal medicine)

(iv) prophylactic treatment: coordinator: Michel Lantéri-Minet (neurologist); participants: Henry Becker (neurologist), Anne Donnet (neurologist), Malou Navez (anaesthetist), Françoise Radat (psychiatrist).

(v) Jean-Christophe Cuvellier (neuropaediatrician) was involved in all areas of migraine in children.

A reading group was set up comprised of members of the SFEMC and independent health professionals (notably community GPs and pharmacists), and members of the patients’ association. Initially, the project was set up at the request of the *Haute Autorité de la Santé* (HAS), but the latter challenged the majority of members of the working group on the grounds of potential conflicts of interest. The SFEMC therefore decided to produce these recommendations in its own name.

## Migraine in adults

### Prevalence

In adults between 18- and 65-years, the prevalence of migraine is estimated to be between 17 and 21% depending on the diagnostic criteria used: strict migraine 8 − 11%, probable migraine 9 − 10%, with a female predominance of 3:1.

### Clinical diagnosis

It is recommended that the diagnostic criteria, established in 1988, revised in 2004 and confirmed in 2013 by the International Headache Society (IHS) on the basis of expert consensus, are used. Only the diagnosis of migraine without aura, typical migraine with aura and probable migraine without aura (satisfying all of the diagnostic criteria except one) are discussed in this document.

The diagnosis of migraine is based on the following clinical triad (professional agreement):

(i) recurrent headache disorder manifesting in attacks

(ii) typical characteristics;

(iii) a normal clinical examination.

The IHS diagnostic criteria for migraine without and with aura are shown below in Tables 1 and 2. These criteria, which are easy to use, enable essential questions to be asked in a logical order and structure. It is recommended that they are used in a systematic way in daily practice (professional agreement).

**Table 1:** Diagnostic criteria for migraine without aura (ICHD-3 beta)

A.	At least five attacks fulfilling to criteria B to D.
B.	Headache attacks lasting 4–72 hours (untreated or unsuccessfully treated).
C.	Headaches has at least two of the following 4 characteristics:
	1- unilateral location
	2- pulsating quality
	3- moderate or severe pain intensity
	4- aggravation by or causing avoidance of routine physical activity (e.g. walking or climbing stairs).
D.	During headache at least one of the following:
	1- nausea and/or vomiting
	2- photophobia and phonophobia.
E.	Not better accounted for by an order ICHD-3 diagnosis

**Table 2:** Diagnostic criteria for migraine with aura (ICHD-3 beta)

A.	At least two attacks responding to criteria B and C.
B.	B. One or more of the following fully reversible aura symptoms:
	1- visual
	2- sensory
	3- speech and/or language
	4- motor
	5- brainstem
	6- retinal
C.	At least two of the following four characteristics:
	1- at least one aura symptom spreads gradually over ≥ 5 minutes, and/or 2 or more symptoms occur in succession
	2- each individual aura symptom last 5–60 minutes
	3- at least one aura symptom is unilateral
	4- the aura is accompanied, or followed within 60 minutes by headache
D.	Not better accounted for by an order ICHD-3 diagnosis, and transient ischemic attack has been excluded

A critical analysis of these criteria shows acceptable inter-observer variability, good specificity, but poor sensitivity. These criteria are therefore restrictive and do not allow the diagnosis of all cases of migraine. In practice, to get around this inconvenience and not deprive some patients with migraine without aura of specific treatment, it is recommended to use the term « probable migraine without aura» for cases that fulfil all of the diagnostic criteria except one. If the five criteria, A, B, C, D and E are present, the diagnosis is migraine without aura in the strict sense of the term. If one of the criteria, A, B, C or D, is not satisfied completely, the diagnosis is probable migraine without aura.

There are three typical symptoms: visual, which are the most frequent (> 90%); sensitive; and aphasic..

A headache occurring after aura may sometimes be of non-migrainous semiology, or even absent (aura without headache). Aura may sometimes occur during the headache. Migraine should be distinguished from a tension headache: more diffuse headache; non pulsatile; not aggravated by effort; less intense; without digestive signs; sometimes accompanied by phonophobia or photophobia but not both at the same time.^1^ A loss of central vision or blurred central vision are possible. Migraine and tension headache may be associated or both occur in the same patient.

Faced with a migraine attack, two misdiagnoses are often allocated:

● « sinusitis » when the pain is frontal or sited around the cheekbone;

● « Arnold’s neuralgia » when the pain starts in the occipital region and spreads forward as migraine.

## Role of complementary examinations

### Cerebral TDM and MRI

It is recommended that all patients – migrainous or not – presenting with a headache of sudden onset, developing in less than 1 min (thunderclap headache), are sent to an emergency department for appropriate complementary examinations.

A CT scan or cerebral MRI is not indicated (professional agreement):

● In a patient with a migraine defined according to IHS criteria for migraine, with or without aura;

● To differentiate a migraine from other primary headaches, in particular a tension headache.

A CT scan or cerebral MRI is recommended (professional agreement):

● In a patient with migraine attacks appearing after the age of 50 years;

● In a patient with atypical aura: sudden onset; lasting for more than 1 h; always occurring on the same side; and/or without visual symptoms;

● An abnormal clinical examination.

In a known migrainous patient, it is recommended that a cerebral scan is performed without injection of a contrast agent in the case of an unusual headache, and if the scan is normal, a cerebral MRI with arterial and venous angioMRI can be performed subsequently, within a period to be determined depending on the context (professional agreement).

### EEG

There is no indication to perform an EEG in a patient with migraine defined according to IHS criteria (professional agreement). EEG is not recommended to eliminate a secondary headache, but cerebral imaging is indicated (professional agreement).Radiography of sinuses, radiography of the neck, ophthalmological examination, orthoptic examination, abdominal echography.

There is no indication to perform radiography of the sinuses, radiography of the neck, an ophthalmological examination, an orthoptic examination, or abdominal echography in the investigation of migraine (professional agreement).

## How do we evaluate the handicap caused by migraine for optimal management?

Migraine is a disabling disease, due to the frequency of attacks (two or more per month in 42 − 50% of patients), their duration (>24 h in 39% of patients), their intensity (severe or very severe in 48 − 74% of patients), the accompanying digestive signs and the alterations in professional, social and familial quality of life.

In order to optimise the management of patients with migraine, it is recommended (professional agreement) that the patient keep a diary of attacks outlining the number of days per month with a migraine headache, the duration and intensity of pain, any triggering factors and all medicines used at each migraine attack (on prescription or not). The diary should also include any headaches that occur in between and their treatments. This tool helps the physician to evaluate the severity of the migraine better, to take into account changes in quality of life, to determine treatment choice and the modalities of follow-up and to detect medication abuse.

The functional repercussions of migraine and changes in productivity can be evaluated using generic and specific scales, which have been validated in French. Among these, the HIT-6 and possibly the MIDAS scale are recommended (professional agreement).

It is recommended that the patient be asked about the presence of mood or anxiety syndromes, because these increase disability and may require specific management. In practice, the HAD scale is proposed to evaluate the emotional component of migraine (professional agreement).

## Pharmaceutical treatments

Migraine is an under-diagnosed disease: in French studies, 40% of migrainous patients have never consulted a doctor about their migraine and 60% ignore their migrainous status and the available treatment options. This state leads to a high level of self-medication.

A study of the therapeutic behaviour of migrainous patients shows an overuse of non-specific analgesics, with several drugs often taken for the same attack and the absence of significant relief two hours after the dose in one case in two. Moreover, it reveals an underuse of specific treatments which, when taken immediately, may be justified in patients having severe attacks or attacks that are not relieved by non-specific treatments.

### Acute treatment of migraine

#### Efficacy of different drugs used for acute treatment

Two types of treatment can be distinguished:

● Non-specific treatments (analgesics and non-steroidal anti-inflammatory drugs (NSAIDs);

● Specific treatments (triptans and ergot derivatives), which, by acting on 5 HT1_B/D_ receptors, inhibit neurogenic inflammation and vasodilation supposed to be the origin of migraine headaches.

### Non-specific treatments for migraine attacks

Proof of efficacy has been demonstrated for the following non-specific treatments:

● The following NSAIDs: naproxen, ibuprofen, ketoprofen and diclofenac (grade A methodology). Ketoprofen has marketing approval (MA) for the « treatment of migraine with or without aura » and ibuprofen has MA for the « treatment of mild to moderate migraine with or without aura »; the other NSAIDs do not have specific MA for the acute treatment of migraine;

● Acetylsalicylic acid (ASS; aspirin) as monotherapy (grade A methodology), or in association with metoclopramide (grade A methodology). Only the association ASS-metoclopramide has MA for the « symptomatic treatment of migraine and associated digestive problems »;

● Paracetamol as monotherapy (grade C methodology). Paracetamol does not have specific MA for the acute treatment of migraine.

The association of metoclopramide with ASS improves digestive symptoms, but does not increase the analgesic effect of ASS (professional agreement). There is no clinical proof that the association of caffeine with paracetamol and aspirin increases their efficacy and this combination cannot be recommended, particularly because caffeine may induce drug abuse (grade B methodology), or even addictive behaviour (professional agreement). It is recommended that opioids are avoided (codeine, opium, tramadol, morphine and other strong opioids), alone or in association, as they may induce drug abuse (grade A methodology), or even addictive behaviour (grade B methodology), and can also increase nausea (grade A methodology).

#### Specific treatments for migraine attacks

Proof of efficacy has been shown for the following specific treatments:

##### Triptans (grade A methodology)

Triptans are effective against headaches, but also against the associated digestive symptoms as well as phonophobia and photophobia (grade A methodology). The following seven triptans have MA for « treatment of the headache phase of migraine »: almotriptan, eletriptan, frovatriptan, naratriptan, rizatriptan, sumatriptan and zolmitriptan.

There are minimal differences in efficacy and tolerance between the triptans (grade A methodology), but in practice there is great interindividual variability (professional agreement). A patient who is a non-responder to a triptan during the first attack may then be a responder (grade A methodology). Before concluding that a triptan is ineffective, it is recommended that it is tested over at least three attacks, except if there is poor tolerance (grade A methodology). A patient who is a non-responder to one triptan may respond to another (grade B methodology).

The association sumatriptan and naproxen sodium is more effective than either of the two drugs taken individually (grade A methodology). Taking a triptan early when the headache is mild is more effective than taking the triptan when the headache is moderate to severe in intensity (grade A methodology).

##### Ergotamine tartrate (grade B methodology)

Ergotamine tartrate associated with caffeine has MA for the « acute treatment of migraine ».

##### Dihydroergotamine (pernasal and injectable) (grade B methodology)

Dihydroergotamine via the pernasal route has MA for the « acute treatment of migraine ».

The acute treatments for migraine attacks with MA are shown in Table 3.

**Table 3:** Medicines with marketing approval (MA) for the acute treatment of migraine attacks

**Active component**	**Dose (per day)**	**Side-effects**	**Contraindications**
***Symptomatic treatment for migraine attacks and for associated digestive problems***			
Lysine acetylsalicylate + metoclopramide	900 mg at the start of the attack	*Linked to metoclopramide*	*Linked to metoclopramide* Pheochromocytoma, gastrointestinal haemorrhage, stenosis or perforation of the gut, previous history of late drug dyskinesia, contra-indicated in children
		Neuropsychiatric problems, late dyskinesia, extrapyramidal syndrome, endocrine problems	
		*Linked to salicylate*	*Linked to salicylate*
		Digestive problems, haemorrhagic syndrome, sensitivity reaction, Reyes syndrome	Gastro-duodenal ulcer, hypersensitivity to salicylates, haemorrhagic risk
***Specific treatments : ergot derivatives***			
Ergotamine tartrate	Adult/child >10 years	Ergotism, nausea, vomiting	Hypersensitivity to ergot derivatives, obstructive coronary artery disease, heart failure, shock, arterial hypertension, severe infection, severe liver failure
	Adult: 2 mg/day (up to 6 mg/day maximum and 10 mg/week maximum).		
	Child >10 years: 1/2 dose		
Dihydroergotamine	Adult >16 years and <65 years	Ergotism, precordialgia with the injectable form, transient local reactions such as nasal obstruction and rhinorrhoea with the endonasal form	
	Endonasal solution		
	One spray in each nostril at the start of the attack		
	Injectable solution		
	1 renewable ampoule, 30 to 60 min later		
	2 mg maximum per day and 8 mg maximum per week		
***Specific treatments: selective 5HT1 receptor agonists (adults from 18 to 65 years)***			
Almotriptan	Tablet of 12.5 mg/maximum 25 mg/day	Vasomotor hot flushes, dizziness, feeling of weakness, asthenia, somnolence, nausea, vomiting, rare cases of heart flutter.	Hypersensitivity, previous history of: myocardial infarction, ischemic heart disease, coronary vasospasm (Prinzmetal angina), peripheral vascular disease, cerebrovascular accident or transitory ischemic accident
Eletriptan	Tablet of 40 mg/maximum 80 mg/day		
Frovatriptan	Tablet of 2.5 mg/maximum 5 mg/day		
Naratriptan	Tablet of 2.5 mg/maximum 5 mg/day		
Rizatriptan	Tablets of 5 and 10 mg, dry powder of 10 mg/maximum 20 mg/day		Patients with severe liver failure
Sumatriptan	Tablet of 50 mg/maximum 300 mg/day SC injection ampoule 6 mg/maximum 12 mg/day. Nasal spray of 10 and 20 mg/maximum 40 mg/day	Moderate or severe hypertension, of pins and needles, sensation of heat, of pressure or of suffocation	Moderate or severe hypertension and in patients with uncontrolled mild hypertension
Zolmitriptan	Tablet of 2.5 mg, orodispersible at 2. mg/maximum 10 mg/day		Association with monoamine oxidase inhibitors (MAOI)

#### Therapeutic strategy for migraine attacks

The following strategy is recommended (professional agreement). During the first consultation, the patient should be asked about his/her usual treatment and the relief provided by this treatment. All acute treatments for attacks taken alone or in association should be evaluated by the response to the following four questions:

When you take your usual treatment:

● Do you have sufficient relief 1 to 2 h after taking this treatment?

● Do you use a single dose of this treatment in the day?

● Is this treatment effective over at least two attacks out of three?

● Is this treatment well-tolerated?

If the patient answers yes to the four questions, it is recommended that acute treatment is not modified.If the patient answers no to at least one of the four questions, it is recommended that a NSAID and a triptan are prescribed on the same prescription.

The patient will first take the NSAID and will keep the triptan as rescue therapy if the migraine is not relieved 1 to 2 h after taking the NSAID. This therapeutic sequence should be assessed after three attacks. If the NSAID is effective over at least two out of three attacks and if it is well-tolerated, this therapeutic sequence should be repeated. If the NSAID is ineffective over at least two out of three attacks, the triptan should be taken as first-line to treat following attacks and the treatment should be reevaluated after three new attacks.

If the triptan used straightaway is ineffective over at least two out of three attacks and is well-tolerated, it is important to first check that the dose of triptan has been taken early (in the hour following the onset of the attack) and if this is not the case, recommend to the patient to retry the triptan by taking it early over three consecutive attacks. If the early dose is ineffective or if it is poorly tolerated, the triptan should be changed and reevaluated by taking it early over three consecutive attacks. Finally, if this strategy is ineffective, the patient should be told to use a NSAID and a triptan taken simultaneously.

Treatment should be adapted to the severity of the digestive signs. Antiemetics are recommended in patients with disabling nausea or vomiting.

For all patients, it is recommended to record the number of days per month when the patient is taking acute treatment, in order to spot overuse, which is frequent in migraine sufferers and may lead to chronic daily headaches. It is recommended that a patient consults as soon as they use a treatment regularly two days of more per week for more than 3 months in view of the possible prescription of prophylactic treatment (professional agreement).

No treatment has proof of efficacy to reduce the duration of aura and the triptans are not effective to prevent headaches when taken at the time of aura (grade B methodology). In the case of a migraine with aura, it is recommended that a NSAID is taken immediately at the start of aura to prevent or limit the subsequent headache (professional agreement) and to wait until the start of the headache before taking a triptan (professional agreement).

### Prophylactic treatment

#### Efficacy of different drugs used as prophylactic treatment

Most drugs proposed as prophylactic treatment for migraine are old molecules which have not been evaluated in controlled therapeutic studies with adequate methodological quality. Taking into account the often weak methodology, the different drugs have been classed in three categories: efficacy demonstrated, probable or doubtful (Table 4):

● Efficacy demonstrated (grade A methodology): valproate and sodium divalproate, metoprolol (MA), propranolol (MA), topiramate (MA);

● Efficacy probable (grade B or C methodology): amitriptyline, atenolol, candesartan, flunarizine (MA), methysergide (MA/recently reevaluated by the Commission de Transparence with an unfavourable benefit/risk ratio), nadolol, naproxen sodium, nebivolol, oxetorone (MA), pizotifen (MA), timolol, venlafaxine;

● Efficacy doubtful (grade B or C methodology): dihydroergotamine (MA), indoramin (MA), gabapentin

**Table 4:** Dosage, side-effects and contraindications for prophylactic treatment

**Active component**	**Dosage (per day)**	**Side-effects**	**Contraindications**
Propranolol	40-240 mg	Frequent: asthenia, poor tolerance to effort	Asthma, heart failure, atrio-ventricular block, bradycardia
Metoprolol	100-200 mg		
Timolol (without MA)	10-20 mg		
Atenolol (without MA)	100 mg	Rare: insomnia, nightmares, impotence, depression	NB: possibility of aggravation of migraines with aura
Nadolol (without MA)	80-240 mg		
Nebivolol (without MA)	5 mg		
Oxetorone	60-180 mg (1–3 tablets) as one dose in the evening	Frequent: somnolence	
		Rare: diarrhoea necessitating discontinuation of treatment	
Amitriptyline	10-50 mg in the evening	Dry mouth, somnolence	Glaucoma, prostatic adenoma
		Weight gain	
Pizotifen	3 tablets per day at progressive doses	Sedation	Glaucoma, uredo-prostatic problems
		Weight gain	
		Rare: digestive problems, dizziness, muscular pain, asthenia	
Topiramate	50-100 mg	Paresthesia	Hypersensitivity to topiramate
		Weight loss	Pregnancy
		Cognitive effects (word-finding difficulties)	
		Rare: renal calculi, acute myopia associated with secondary angle closure glaucoma	
Sodium valproate (without MA)	500-1000 mg	Nausea, weight gain, somnolence, trembling, alopecia, liver attack	Liver diseases
			Pregnancy
Methysergide	2-6 mg (1–3 tablets) Necessary to stop treatment for 1 month every 6 months	Frequent: nausea, dizziness, insomnia	Hypertension, heart failure, arteriopathologies, gastric ulcer, liver and kidney failure, association with triptans
		Rare: retroperitoneal fibrosis	
Flunarizine	10 mg (1 tablet in the evening). Not for more than 6 consecutive months	Frequent: somnolence, weight gain	Depressive syndrome, extra-pyramidal syndrome
		Rare: depression, extrapyramidal syndrome	
Gabapentin (without MA)	1200-2400 mg	Nausea, vomiting, convulsions, somnolence, ataxia, dizziness	Hypersensitivity to gabapentin
Dihydroergotamine	10 mg	Nausea	Association with triptans
Indoramin	50 mg	Somnolence, nasal congestion, dry mouth, ejaculation problems	Hypersensitivity to one of the components of the drug product, Parkinson’s disease, severe heart, liver and kidney failure
Candesartan (without MA)	8-16 mg	Arterial hypotension, dizziness	Hypersensitivity, severe liver and kidney failure
			2nd and 3rd trimester of pregnancy
Venlafaxin (without MA)	75-150 mg	Nausea, dizziness, hypersudation, somnolence, nervousness, dry mouth	Hypersensitivity to venlafaxine, association with non-selective MAOI, congenital galactosaemia, breast feeding

The age of these drugs explains the absence of correlation between level of proof and MA. Thus, the following have MA as prophylactic treatment for migraine: dihydroergotamine, flunarizine, indoramin, metoprolol, methysergide, oxetorone, pizotifen, propranolol, topiramate. In addition to the level of proof and MA, the strategy in terms of prophylaxis is also determined by the benefit/risk ratio (professional agreement). No drug has been shown to have superior efficacy compared to the others (grade B methodology).

#### Therapeutic strategy for prophylactic treatment (professional agreement)

This strategy depends on a number of questions which the prescriber faces.

##### When should prophylactic treatment be started?

It is recommended that prophylactic treatment is started:

● As a function of the frequency and intensity of attacks, but also the familial, social and professional handicap caused by the attacks;

● As soon as the patient uses treatment(s) for attacks more than 2 days each week, for 3 months, even in the case of efficacy, in order to avoid drug abuse.

The initiation of prophylactic treatment should be associated with an educational strategy in which it should be explained to the patient that prophylactic treatment will not prevent attacks but will reduce their frequency and intensity. Keeping a diary of attacks will allow a better appreciation of the efficacy of prophylactic treatment.

##### Which drugs should be used as prophylactic treatment?

Considering the level of proof of efficacy, the benefit/risk ratio and the existence of MA, the preferred drugs to be used as prophylaxis are propranolol and metoprolol, in the absence of a contraindication to the use of betablockers. In the case of a contraindication, intolerance or inefficacy of these betablockers, the choice of drug depends on the context, including comorbidities and migraine severity, whilst also considering the benefit/risk ratio (weight gain, sedation, asthenia and teratogenic risk) and the existence of MA.

##### How should prophylactic treatment be started?

It is recommended that prophylactic treatment is started as monotherapy and at a low dose, and that the dose is increased progressively to achieve the optimal dose, taking into account possible side-effects.

##### How should prophylactic treatment be evaluated?

Prophylactic treatment is considered to be effective when it reduces the frequency of attacks by at least 50%. It is important to also take into account the decrease in consumption of acute treatments, and the intensity and duration of attacks. Effectiveness should be evaluated after three months. In the case of failure, there are two possibilities:

● The dose can be increased, in the absence of side-effects;

● Another treatment may be proposed.

The association of two prophylactic treatments at a lower dose may be envisaged with the aim of reducing the side-effects of each drug, after having tested them separately. In the case of repeated failures, compliance or drug abuse should be investigated.

##### When should prophylactic treatment be stopped?

In the case of success, prophylactic treatment at the effective dose should be continued for six months to one year, adapted as closely as possible to the spontaneous evolution of migraine and then decreased very slowly before being stopped. The same treatment may be restarted if the frequency of attacks increases again.

## Other treatments

Relaxation, retrocontrol (biofeedback) and cognitive and behavioural therapies for the management of stress have proof of efficacy (grade A methodology) and may be recommended. Data in the literature are inconclusive about the efficacy of acupuncture (grade A methodology), but do not recommend homeopathy (grade A methodology) or spinal manipulation (professional agreement) for the prevention of migraine.

## Characteristics of migraine in children

### Prevalence

The prevalence is estimated to be between 3 and 10%.

### Positive diagnosis

Migraine in children can be distinguished from adult migraine by:

● Shorter attacks (1–48 h in children <15-years according to the IHS);

● A more frequent bilateral localisation;

● Digestive problems are often more important;

● Frequent initial pallor.

As in adults, it is recommended to use the diagnosis of «probable migraine without aura» when all diagnostic criteria are fulfilled except one, so as not to deprive some children of specific management. In this context, the IHS criteria for the diagnosis of migraine without aura have a lower sensitivity in children than in adults.

### Place of complementary examinations

The place of complementary examinations is the same in children as in adults. However, the indications for neuroimaging should be extended due to the difficulties in the aetiological diagnosis of headaches in children.

### Evaluation of handicap

No quality of life scale has been validated in French for migraine in children. It is recommended to keep a diary of attacks in order to help the child and his/her family identify triggering factors, to evaluate the efficacy of treatments and to allow the doctor to appreciate the severity of the migraine (frequency, intensity of attacks, associated digestive signs) and its repercussions on daily life (absenteeism from school).

### Acute treatment of migraine attacks

The following drugs are recommended in children and adolescents as first-line:

● Ibuprofen in children >6 months (grade A methodology);

● Then (professional agreement): diclofenac in children >16 kg, naproxen in children >6 years or >25 kg, aspirin as monotherapy, paracetamol as monotherapy.

In the treatment of moderate to severe migraine attacks, sumatriptan nasal spray (10–20 mg) is effective (grade A methodology) and has specific MA in adolescents from 12 to 17 years. It is recommended (professional agreement):

● To take treatment as early as possible;

● To use the rectal route in the case of nausea and vomiting;

● To use the nasal route from 12-years of age or in children >35 kg;

● To use sumatriptan nasal spray in the case of failure with paracetamol, aspirin and NSAIDs;

● For the triptans and ergot derivatives, to wait for the onset of the headache to treat an attack with aura.

### Prophylactic treatment

#### Non-pharmaceutical treatments

Relaxation, retrocontrol (biofeedback) and cognitive and behavioural therapies for the management of stress can be recommended (grade B methodology). These treatments are more effective than betablockers (grade B methodology).

#### Pharmaceutical treatments

It is recommended that prophylactic drug treatment is used after failure of non-pharmacological treatments (professional agreement), although none of these treatments has MA in this paediatric indication. In the absence of established scientific proof, the following drugs may be proposed, in no preferential order (professional agreement):

● Amitriptyline, 3–10 mg/day;

● Flunarizine in children >10 years, 5 mg/day;

● Metoprolol, 25–50 mg/day;

● Oxetorone, 15–30 mg/day;

● Pizotifen in children >12 years, 1 mg/day;

● Propranolol, 2–4 mg/kg/day;

● Topiramate, 50–100 mg/day.

It is recommended to use these drugs at low doses, in order to limit the side-effects, particularly their sedative effects.

## Migraine and the hormonal cycle in women

### Migraine and pregnancy

#### Management of migraine in a woman desiring a pregnancy

Confronted with a female migraine sufferer who wishes to become pregnant, the following recommendations can be made regarding the planning of migraine treatment:

● Reassure her that, as far as it is known, migraine is not associated with a poor evolution of pregnancy (grade A methodology);

● Reassure her by indicating that for the great majority of women with migraine, pregnancy is associated with a partial or even complete remission of migraine attacks (grade A methodology);

● Do not start prophylactic treatment (professional agreement);

● According to recommendations suggest acute treatment favouring paracetamol, but not limiting the use of aspirin, NSAIDs and triptans (ideally favouring triptans in the first 2 weeks of the cycle and aspirin or NSAIDs during the rest of the cycle) (professional agreement);

● Inform her about the antimigraine drugs that are contraindicated during pregnancy (ergot derivatives, valproate and sodium divalproate; aspirin and NSAIDs from the end of the fifth month of pregnancy) (professional agreement);

● Remind her of the risk of some drugs (notably ibuprofen) and phytotherapeutics available in the pharmacy or parapharmacy without prescription (professional agreement);

● Inform her that migraine can be treated during her pregnancy, if necessary, and that breast feeding (which is advised) will be possible (professional agreement).

#### Steps to take in a female migraine sufferer who has used antimigraine drugs unaware that she was pregnant

A woman with migraine may seek advice because she has just become pregnant and has used antimigraine drugs when she did not know that she was pregnant.

##### Taking antimigraine drugs prophylactically when she did not know that she was pregnant

For most of the prophylactic antimigraine drugs, it is sufficient to reassure the patient and inform them that no surveillance of the pregnancy is necessary (except if the patient has taken prophylaxis with ergot derivatives [DHE or methysergide] or valproate or sodium divalproate) (professional agreement). In the case of prophylactic treatment with a drug belonging to the class of betablockers (propranolol and metoprolol) or with tricyclic antidepressants (amitriptyline), this drug should be stopped immediately bearing in mind that if it is justified by the migraine, this treatment could be continued at the minimal effective dose (professional agreement).

In the case of prophylactic treatment with a drug that is neither a betablocker nor a tricyclic, the drug should be stopped. If prophylactic treatment is justified however, the drug should be replaced by a betablocker (propranolol or metoprolol) or possibly by a tricyclic (amitriptyline) (professional agreement).

##### Taking acute antimigraine treatment when the patient does not know that she is pregnant

For most antimigraine drugs used as acute treatment for attacks, it is advisable to reassure the patient and inform them that no surveillance of the pregnancy is necessary (except if the patient has used large quantities of DHE or ergotamine tartrate) (professional agreement).

Concerning the continuation of a drug during pregnancy, the advice will depend on the drug concerned (professional agreement):

● Paracetamol: its use as first-line is possible;

● Aspirin and NSAIDs: paracetamol is preferred as first-line, but these drugs can be used as rescue therapy during the second and third trimesters, while they are contraindicated from the end of the fifth month;

● DHE and ergotamine tartrate: their use is positively contraindicated;

● Triptans: although pharmacovigilance data are reassuring, their use is contraindicated.

In all cases, exposure of the patient to drugs should be declared to pharmacovigilance (pharmacovigilance unit of the hospital, pharmacovigilance department of the company producing the drug concerned, reference centre for teratogenic agents [http://www.lecrat.org]).

#### Recommendations for the management of migraine in pregnancy when treatment is necessary

A number of recommendations allow treatment in a pregnant migraine sufferer to be optimised if necessary. These recommendations are as follows:

● Plan monthly follow-up visits when remission from attacks is not observed (professional agreement);

● Propose acute treatment with paracetamol as first-line and a NSAID as rescue therapy (only during the first and second trimesters because after this time aspirin and NSAIDs are contraindicated) (professional agreement);

● If prophylactic treatment is necessary, favour a betablocker (propranolol or metoprolol) or as second-line a tricyclic antidepressant (amitriptyline) (reminding the patient that it is necessary to stop these drugs before delivery) (professional agreement);

● Remind the patient about the risks of these drugs (ibuprofen in particular) and of the phytotherapeutic preparations available from the pharmacy or parapharmacy without a prescription (professional agreement).

In all cases where a drug is prescribed, it is necessary to remember that exposure of the patient to the drug should be declared to pharmacovigilance (pharmacovigilance unit of the hospital, pharmacovigilance department of the company producing the drug concerned, reference centre for teratogenic agents: http://www.lecrat.org).

### Catamenial (menstrual) migraine

According to the international classification of headaches by the IHS (ICHD-III beta), the diagnosis of menstrual migraine depends on the appearance, during at least two out of three consecutive menstrual cycles, of an attack without aura starting between the second day before and the third day following the menstrual period, whether this menstrual period corresponds to natural menstruation or withdrawal bleeding following the discontinuation of oral oestroprogestative contraception. The diagnosis of catamenial migraine (or purely menstrual migraine) is made in migraine sufferers who do not cite any other attack outside the menstrual period.

Although nearly half of migraine sufferers report menstrual attacks, less than 10% report a catamenial migraine (grade B methodology).

Monthly attacks are secondary to the fall in oestrogens occurring during the luteal phase of the natural menstrual cycle or during the discontinuation of oral oestroprogestative contraception (grade B methodology). Catamenial migraine indicates a particular sensitivity to these hormonal variations in women who suffer from them.

Compared to attacks occurring outside the menstrual period, menstrual attacks are characterised by a greater severity, a longer duration and a poorer response to acute treatment (grade A methodology). These attacks may have serious repercussions as some patients may suffer anxious anticipation, the time in the menstrual cycle drives them to «anticipate» the appearance of monthly menstrual attack (professional agreement).

Menstrual migraine attacks should be treated in the same way as migraine attacks occurring outside the menstrual period (professional agreement).

In patients suffering from catamenial migraine and if acute treatment is not effective, sequential prophylactic treatment may be considered, that is to say, limited to the menstrual period. Several options can be considered knowing that none have specific MA in this indication. It is possible to use cutaneous oestradiol at a dose of 1.5 mg/day for 7 days starting on the second day before the menstrual period or withdrawal bleeding (grade B methodology). More recently, some triptans have been shown to be effective as prophylactic sequential therapy: frovatriptan at a daily dose of 2.5 mg two times/day (grade A methodology), naratriptan at a dose of 1 mg two times/day (grade B methodology) and zolmitriptan at a dose of 2.5 mg two times/day (grade B methodology). In patients taking oral contraception, menstrual migraine can be prevented by using a continuous oestroprogestative or a pure progestative (professional agreement).

### Migraine and oral contraception

The use of oral contraception in a migraine sufferer should be considered carefully bearing two questions in mind:

● Is there a risk that the contraception will aggravate the migraine?

● Will the contraception expose the patient to a particular vascular risk?

The association between the prevalence of migraine and past or present use of oral contraception is linked to the presence of ethinyl-oestradiol (independent of the dose) but not to that of the progestative (grade A methodology). In spite of this association, the influence of contraception on migraine is subject to great inter-individual variability; thus, oral contraception is not in principal contraindicated in women with migraine (professional agreement).

Young patients (<35 years) suffering from migraine with aura have an increased neurovascular risk (grade A methodology). This neurovascular risk is increased in the presence of cofactors, particularly smoking and the use of oestroprogestative oral contraception (grade A methodology). In young migraine sufferers with aura, particularly when they smoke, oestroprogestative oral contraception is contraindicated and oral contraception which is purely progestative or another means of contraception should be preferred (professional agreement).

### Migraine and hormonal treatment of the menopause (HRT)

The use of hormone replacement therapy (HRT) in a woman with migraine should be considered bearing two questions in mind:

● Is there a risk that HRT will have an influence on the migraine?

● Is there a risk of cerebral ischaemia from HRT in the migrainous patient?

#### Influence of HRT on the course of migraine

Transversal studies (grade B methodology) have demonstrated a significant association between HRT use and persistence of migraine attacks. Longitudinal studies (grade B methodology) have shown that transdermal oestradiol induces fewer migraines than oral conjugated oestrogens and that HRT taken continuously induces fewer migraine attacks than discontinuous treatment.

#### HRT, migraine and the risk of cerebral infarction

HRT is an independent risk factor for cerebral infarction with a low but significant relative risk (RR = 1.29; 95%CI: 1.06 − 1.56) as shown in a meta-analysis (grade A methodology). No data are available on the risk associatied with both migraine and HRT combined.

Overall, migraine is not a contraindication for HRT, but if aggravation of migraine is observed, notably with aura on HRT, alternatives should be discussed with the patient, including a change to a transdermal form, a reduction of the oestradiol dose, or stopping HRT completely.

## Future developments

Taking into account the current clinical developments (such as CGRP receptor antagonists and other non-vasoconstrictive treatments), the recommendations of the working group should be amended when necessary during the next five years. Future actions should also be adapted to the recommendations for patients.

## References

1. Aegidius K, Zwart JA, Hagen K, Schei B, Stovner LJ (2006) Oral contraceptives and increased headache prevalence. Neurology 66:349–353

2. Aegidius KL, Zwart JA, Hagen K, Schei B, Stovner LJ (2007) Hormone replacement therapy and headache prevalence in postmenopausal women. The Head-HUNT study. Eur J Neurol 14:73–78

3. Akyol A, Kiylioglu N, Aydin I, Erturk A, Kaya E, Telli E, Akyildiz U (2007) Epidemiology and clinical characteristics of migraine among school children in the Menderes region. Cephalalgia 27:781–787

4. ANAES (2003) Prise en charge diagnostique et thérapeutique de la migraine chez l'adulte et chez l'enfant: aspects cliniques et économiques. Recommandations. Rev Neurol (Paris) 159(6–7 Pt 2):S5–15

5. Anttila P, Metsâhonkala L, Sillanpââ M (2006) Long-term trends in the incidence of headache in Finnish schoolchildren. Pediatrics 117:e1197–1201

6. Arny JJ, Tripathi V (2009) Contraception for women: an evidence based overview. Br Med J 339:563–568

7. Ashcroft DM, Millson D (2004) Naratriptan for the treatment of acute migraine: meta-analysis of randomised controlled trials. Pharmacoepidemiol Drug Saf 13(2):73–82

8. Ashtart F, Shaygannejad V, Akbari M (2008) A double-blind, randomized trial of low-dose topiramate vs propranolol in migraine prophylaxis. Acta Neurol Scand 118:301–305

9. Atkinson MJ, Sinha A, Hass SL, Colman SS, Kumar RN, Brod M, Rowland CR (2004) Validation of a general measure of treatment satisfaction, the Treatment Satisfaction Questionnaire for Medication (TSQM), using a national panel study of chronic disease. Health Qual Life Outcomes 26:2–12

10. Bath PM, Gray LJ (2005) Association between hormone replacement therapy and subsequent stroke: a meta-analysis. BMJ 330:342

11. Bigal ME, Bordini CA, Tepper SJ, Speciali JG (2002) Intravenous magnesium sulphate in the acute treatment of migraine without aura and migraine with aura. A randomized, double-blind, placebo-controlled study. Cephalalgia 22:345–353

12. Bigal ME, Rapoport AM, Lipton RB, Tepper SJ, Sheftell FD (2003) Assessment of migraine disability using the migraine disability assessment (MIDAS) questionnaire: a comparison of chronic migraine with episodic migraine. Headache 43:336–342

13. Bigal ME, Lipton RB, Winner P, Reed ML, Diamond S, Stewart WF (2007) AMPP advisory group Migraine in adolescents: association with socioeconomic status and family history. Neurology 69:16–25

14. Bjomer JB, Kosinski M, Ware JE, Jr (2003) Using item response theory to calibrate the Headache Impact Test (HIT) to the metric of traditional headache scales. Qual Life Res 12:981–1002

15. Brandes JL (2006) The influence of estrogen on migraine. JAMA 295:1824–1830

16. Brandes JL, Saper JR, Diamond M, Couch JR, Lewis DW, Schmitt J, Neto W, Schwabe S, Jacobs D; MIGR-002 Study Group (2004) Topiramate for migraine prevention: a randomized controlled trial. JAMA 291:965–973

17. Brandes JL, Visser WH, Farmer MV, Schuhl AL, Malbecq W, Vrijens F, Lines CR, Reines SA, Protocol 125 study group (2004) Montelukast for migraine prophylaxis: a randomized, double-blind, placebo controlled study. Headache 44:581–586

18. Brandes JL, Kudrow D, Cady R, Tiseo PJ, Sun W, Sikes CR (2005) Eletriptan in the early treatment of acute migraine: Influence of pain intensity and time of dosing. Cephalalgia 25:735–742

19. Brandes JL, Kudrow D, Stark SR, O'Carroll CP, Adelman JU, O'Donnell FJ, Alexander WJ, Spruill SE, Barrett PS, Lener SE (2007) Sumatriptan-naproxen for acute treatment of migraine: a randomized trial. JAMA 297:1443–1454

20. Brandes JL, Poole A, Kallela M, Schreiber CP, MacGregor EA, Silberstein SD, Tobin J, Shaw R (2009) Short-term frovatriptan for the prevention of difficult-to-treat menstruel migraine attacks. Cephalalgia 29:1133–1148

21. Bulut S, Berilgen MS, Baran A, Tekatas A, Atmaca M, Mungen B (2004) Venlafaxine versus amitriptyline in the prophylactic treatment of migraine: randomized, doubleblind, crossover study. Clin Neurol Neurosurg 107:44–48

22. Bushnell CD, Jamison M, James AH (2009) Migraines during pregnancy linked to stroke and vascular diseases: US population based case–control study. Br Med J 338:b664

23. Cady R, Elkind A, Goldstein J, Keywood C (2004) Randomized, placebocontrolled comparison of early use of frovatriptan in a migraine attack versus dosing after the headache has become moderate or severe. Curr Med Res Opin 20:1465–1472

24. Cady R, Martin V, Mauskop A, Rodgers A, Hustad CM, Ramsey KE, Skobieranda F (2006) Efficacy of rizatriptan 10 mg administered early in a migraine attack. Headache 46:914–924

25. Canadian Headache Society Prophylactic Guidelines Development Group (2012) Canadian headache society guideline for migraine prophylaxis. Can J Neurol Sci 39(2 Suppl. 2):1–62

26. Carpay J, Schoenen J, Ahmad F, Kinrade F, Boswell D (2004) Efficacy and tolerability of sumatriptan tablets in a fast disintegrating, rapid-release formulation for the acute treatment of migraine: results of a multicenter, randomized, placebo-controlled study. Clin Ther 26:214–223

27. Cete Y, Dora B, Ertan C, Ozdemir C, Oktay C (2005) A randomized prospective placebo-controlled study of intravenous magnesium sulphate vs. Metoclopramide in the management of acute migraine attacks in the emergency department. Cephalalgia 25:199–204

28. Charlesworth BR, Dowson AJ, Purdy A, Becker WJ, Boes-Hansen S, Farkkila M (2003) Speed of onset and efficacy of zolmitriptan nasal spray in the acute treatment of migraine: a randomised, double-blind, placebo-controlled, doseranging study versus zolmitriptan tablet. CNS Drugs 17:653–667

29. Colman I, Brown MD, Innes GD, Grafstein E, Roberts TE, Rowe BH (2004) Parenteral metoclopramide for acute migraine: metaanalysis of randomised controlled trials. BMJ 329:1369–1373

30. De Diego EV, Lanteri-Minet M (2005) Recognition and management of migraine in primary care: influence of functional impact measured by the headache impact test (HIT). Cephalalgia 25:184–190

31. Demarin V, Vukovic V, Lovrencic-Huzjan A, Lusić I, Janculjak D, Wilheim K, Zurak N (2008) Evidence based guidelines for the treatment of primary headaches. Acta Med Croatica 62:99–136

32. Diamond S, Bigal ME, Silberstein S, Loder E, Reed M, Lipton RB (2007) Pattems of diagnosis and acute and preventive treatment for migraine in the United States: results from the American Migraine Prevalence and Prevention study. Headache 47:355–363

33. Diener HC, Bussone G, De Liano H, Eikermann A, Englert R, Floeter T, Gallai V, Lel H, Hartung E, Jimenez MD, Lange R, Manzoni GC, Mueller Schwefe G, Nappi G, Pinessi L, Prat J, Puca FM, Titus F, Voelker M, EMSASI Study Group (2004) Placebocontrolled comparison of effervescent acetylsalicylic acid, sumatriptan and ibuprofen in the treatment of migraine attacks. Cephalalgia 24:947–954

34. Diener HC, Eikermann A, Gessner U, Gübel H, Haag G, Lange R, May A, Müller-Schwefe G, Voelker M (2004) Efficacy of 1,000 mg effervescent acetylsalicylic acid and sumatriptan in treating associated migraine symptoms. Eur Neurol 52:50–56

35. Diener HC, Gendolla A, Gebert I, Beneke M (2005) Almotriptan in migraine patients who respond poorly to oral sumatriptan: a double-blind, randomized trial. Headache 45:874–882

36. Diener HC, Pfaffenrath V, Schnitker J, Friede M, Henneicke-von Zepelin HH (2005) Efficacy and safety of 6.25 mg tid feverfew CO2- extract (MIG-99) in migraine prevention - a randomized, double-blind, multicentre, placebo-controlled study. Cephalalgia 25:1031–1041

37. Diener HC, Montagna P, Gacs G, Lyczak P, Schumann G, Zoller B, Mulder LJ, Siegel J, Edson K (2006) Efficacy and tolerability of diclofenac potassium sachets in migraine: a randomized, double-blind, cross-over study in comparison with diclofenac potassium tablets and placebo. Cephalalgia 26:537–547

38. Diener HC, Gendolla A, Feuersenger A, Evers S, Straube A, Schumacher H, Davidai G (2009) Telmisartan in migraine prophylaxis: a randomized, placebo-controlled trial. Cephalalgia 29:921–927

39. Diener HC, Tfelt Hansen P, Dahlöf C, Lainez MJ, Sandrini G, Wang SJ, Neto W, Vijapurkar U, Doyle A, Jacobs D (2004) MIGR-003 Study Group (2004) Topiramate in migraine prophylaxis - results from a placebo-controlled trial with propranolol as an active control. J Neurol 251:943–950

40. Dodick DW, Papademetriou V (2004) Cardiovascular safety of triptans. Cephalalgia 24:513–514

41. Dodick D, Brandes J, Elkind A, Mathew N, Rodichok L (2005) Speed of onset, efficacy and tolerability of zolmitriptan nasal spray in the acute treatment of migraine: a randomised, double-blind, placebo-controlled study. CNS Drugs 19:125–136

42. Dodick DW, Freitag F, Banks J, Saper J, Xiang J, Rupnow M, Biondi D, Greenberg SJ, Hulihan J, CAPSS-277 Investigator Group (2009) Topiramate versus amitriptyline in migraine prevention: a 26-wk, multicenter, randomized, doubleblind, double-dummy, parallel-group noninferiority trial in adult migraineurs. Clin Ther 31:542–559

43. Dowson AJ, Massiou H, Lainez JM, Cabarrocas X (2004) Almotriptan improves response rates when treatment is within 1 hour of migraine onset. Headache 44:318–322

44. Duru G, Auray JP, Gaudin AF, Dartigues JF, Henry P, LantériMinet M, Lucas C, Pradalier A, Chazot G, El Hasnaoui A (2004) Impact of headache on quality of life in a general population survey in France (GRIM2000 Study). Headache 44:571–580

45. Eftedal OS, Lydersen S, Helde G, White L, Brubakk AO, Stovner LJ (2004) A randomized, double blind study of the prophylactic effect of hyperbaric oxygen therapy on migraine. Cephalalgia 24:639–644

46. El Hasnaoui A, Vray M, Richard A, Nachit Ouinekh F, Boureau F, MIGSEV Group (2003) Assessing the severity of migraine: development of the MIGSEV scale. Headache 43:628–635

47. Etminan M, Takkouche B, Isoma FC, Samii A (2005) Risk of ischaemic stroke in people with migraine: systematic review and meta-analysis of observational studies. Br Med J 330:63

48. Evers S, Afra J, Frese A, Goadsby PJ, Linde M, May A, Sándor PS, European Federation of Neurological Societies (2009) EFNS guideline on the drug treatment of migraine - revised report of an EFNS task force. Eur J Neurol 16:968–981

49. Facchinetti F, Nappi RE, Tirelli A, Polatti F, Nappi G, Sances G (2002) Hormone supplementation differently affects migraine in postmenopausal women. Headache 42:924–929

50. Farkkila M, Olesen J, Dahlof C, Stovner LJ, ter Bruggen JP, Rasmussen S, Muirhead N, Sikes C (2003) Eletriptan for the treatment of migraine in patients with previous poor response or tolerance to oral sumatriptan. Cephalalgia 23:463–471

51. Fendrich K, Vennemann M, Pfaffenrath V, Evers S, May A, Berger K, Hoffmann W (2007) Headache prevalence among adolescents - the German DMKG headache study. Cephalalgia 27:347–354

52. Foley KA, Cady R, Martin V, Adelman J, Diamond M, Bell CF, Dayno JM, Hu XH (2005) Treating early versus treating mild: Timing of migraine prescription medications among patients with diagnosed migraine. Headache 45:538–545

53. Freitag FG, Finlayson G, Rapoport AM, Elkind AH, Diamond ML, Unger JR, Fisher AC, Armstrong RB, Hulihan JF, Greenberg SJ (2007) Effect of pain intensity and time to administration on responsiveness to almotriptan: results from axert 12.5 mg time versus intensity migraine study (aims). Headache 47:519–530

54. Freitag F, Smith T, Mathew N, Rupnow M, Greenberg S, Mao L, Finlayson G, Wright P, Biondi D (2008) Effect of early intervention with almotriptan vs placebo on migraine-associated functional disability: results from the aegis trial. Headache 48:341–354

55. Garcia-Ramos G, MacGregor EA, Hilliard B, Bordini CA, Leston J, Hettiarachchi J (2003) Comparative efficacy of eletriptan vs. Naratriptan in the acute treatment of migraine. Cephalalgia 23:869–876

56. Gawel M, Aschoff J, May A, Charlesworth BR (2005) Zolmitriptan 5 mg nasal spray: Efficacy and onset of action in the acute treatment of migraine - results from phase 1 of the realize study. Headache 45:7–16

57. Geraud G, Compagnon A, Rossi A (2002) Zolmitriptan versus a combination of acetylsalicylic acid and metoclopramide in the acute oral treatment of migraine: A double-blind, randomised, three-attack study. Eur Neurol 47:88–98

58. Gerbaud L, Navez ML, Couratier P, Lejeune ML, Vernay D, Aufauvre D, Preux PM, Metz O, Dazord A, Laurent B, Clavelou P (2002) Validation of the combined SF36/MSQ0L test of evaluation of quality of life in migraine patients in France. Rev Neurol 158:719–727

59. Goadsby PJ, Zanchin G, Geraud G, de Klippel N, Diaz-Insa S, Gobel H, Cunha L, Ivanoff N, Falques M, Fortea J (2008) Early vs. Non-early intervention in acute migraine-"act when mild (awm)". A double-blind, placebo-controlled trial of almotriptan. Cephalalgia 28:383–391

60. Goadsby PJ, Goldberg J, Silberstein SD (2008) Migraine in pregnancy. Br Med J 336:1502–1504

61. Gobel H, Heinze A, Niederberger U, Witt T, Zumbroich V (2004) Efficacy of phenazone in the treatment of acute migraine attacks: a double-blind, placebo-controlled, randomized study. Cephalalgia 24:888–893

62. Goldstein J, Silberstein SD, Saper JR, Elkind AH, Smith TR, Gallagher RM, Battikha JP, Hoffman H, Baggish J (2005) Acetaminophen, aspirin, and caffeine versus sumatriptan succinate in the early treatment of migraine: results from the asset trial. Headache 45:973–982

63. Goldstein LB, Bushnell CD, Adams RJ, Appel LJ, Braun LT, Chaturvedi S, Creager MA, Culebras A, Eckel RH, Hart RG, Hinchey JA, Howard VJ, Jauch EC, Levine SR, Meschia JF, Moore WS, Nixon JV, Pearson TA, American Heart Association Stroke Council; Council on Cardiovascular Nursing; Council on Epidemiology and Prevention; Council for High Blood Pressure Research, Council on Peripheral Vascular Disease, and Interdisciplinary Council on Quality of Care and Outcomes Research (2011) Guidelines for the primary prevention of stroke. A guideline for healthcare professionals from the American Heart Association/American Stroke Association. Stroke 42:517–584

64. Granella F, Sances G, Allais G, Nappi RE, Tirelli A, Benedetto C, Brundu B, Facchinetti F, Nappi G (2004) Characteristics of menstrual and nonmenstrual attacks in women with menstrually related migraine referred to headache centres. Cephalalgia 24:707–716

65. Gupta P, Singh S, Goyal V, Shukla G, Behari M (2007) Low dose topiramate versus lamotrigine in migraine prophylaxis (the Lotolamp study). Headache 47:402–412

66. Harris M, Kaneshiro B (2009) An evidence-based approach to hormonal contraception and headaches. Contraception 80:417–421

67. Headache Classification Subcommittee of the International headache Society (2004) The International Classification of Headache Disorders: 2nd revision. Cephalalgia 24(Suppl. 1):9–160

68. Henry P, Auray JP, Gaudin AF, Dartigues JF, Duru G, LantériMinet M, Lucas C, Pradalier A, Chazot G, El Hasnaoui A (2002) Prevalence and clinical characteristics of migraine in France. Neurology 59:232–237

69. Hershey AD, Powers SW, Vockell AL, Le Cates S, Kabbouche MA, Maynard MK (2001) PedMIDAS: development of a questionnaire to assess disability of migraines in children. Neurology 57:2034–2039

70. Jelinski SE, Becker WJ, Christie SN, Ahmad FE, Pryse-Phillips W, Simpson SD (2006) Pain free efficacy of sumatriptan in the early treatment of migraine. Can J Neurol Sci 33:73–79

71. Kallen B, Lygner PE (2001) Delivery outcome in women who used drugs during pregnancy with special reference to sumatriptan. Headache 41:351–356

72. Karli N, Akis N, Zarifoglu M, Akgôz S, Irgil E, Ayvacioglu U, Calisir N, Haran N, Akdogan O (2006) Headache prevalence in adolescents aged 12 to 17: a student-based epidemiological study in Bursa. Headache 46:649–455

73. Keskinbora K, Aydinli I (2008) A double-blind randomized controlled trial of topiramate and amitriptyline either alone or in combination for the prevention of migraine. Clin Neurol Neurosurg 110:979–984

74. Klapper J, Lucas C, Rosjo O, Charlesworth B (2004) Benefits of treating highly disabled migraine patients with zolmitriptan while pain is mild. Cephalalgia 24:918–924

75. Kolodny A, Polis A, Battisti WP, Johnson-Pratt L, Skobieranda F (2004) Comparison of rizatriptan 5 mg and 10 mg tablets and sumatriptan 25 mg and 50 mg tablets. Cephalalgia 24:540–546

76. Kurth T (2007) Migraine with aura and ischaemic stroke: which additional factors matter? Stroke 38:2407–2409

77. Landy S, Savani N, Shackelford S, Loftus J, Jones M (2004) Efficacy and tolerability of sumatriptan tablets administered during the mild-pain phase of menstrually associated migraine. Int J Clin Pract 58:913–919

78. Lantéri-Minet M, Auray JP, El Hasnaoui A, Dartigues JF, Duru G, Henry P, Lucas C, Pradalier A, Chazot G, Gaudin AF (2003) Prevalence and description of chronic daily headache in the general population in France. Pain 02:143–149

79. Leinisch E, Evers S, Kaempfe N, Kraemer C, Sostak P, Jurgens T, Straube A, May A (2005) Evaluation of the efficacy of intravenous acetaminophen in the treatment of acute migraine attacks: a double-blind, placebo-controlled parallel group multicenter study. Pain 117:396–400

80. Levy MJ, Mathan MS, Bhola R, Meeran K, Goadsby PJ (2005) Octreotide is not effective in the acute treatment of migraine. Cephalalgia 25:48–55

81. Lima MM, Padula NA, Santos LC, Oliveira LD, Agapejev S, Padovani C (2005) Critical analysis of the international classification of headache disorders diagnostic criteria (ICHD I-1988) and (ICHD II-2004), for migraine in children and adolescents. Cephalalgia 25:1042–1047

82. Lipton RB, Gobel H, Einhaupl KM, Wilks K, Mauskop A (2004) Petasites hybridus root (butterbur) is an effective preventive treatment for migraine. Neurology 63:2240–2244

83. Lipton RB, Goldstein J, Baggish JS, Yataco AR, Sorrentino JV, Quiring JN (2005) Aspirin is efficacious for the treatment of acute migraine. Headache 45:283–292

84. Lipton RB, Bigal ME, Diamond M, Freitag F, Reed ML, Stewart WF, The American Migraine Prevalence and Prevention Advisory Group (2007) Migraine prevalence, disease burden, and the need for preventive therapy. Neurology 68:343–349

85. Loder E, Silberstein SD, Abu-Shakra S, Mueller L, Smith T (2004) Efficacy and tolerability of oral zolmitriptan in menstrually associated migraine: a randomized, prospective, parallelgroup, double-blind, placebo-controlled study. Headache 44:120–130

86. Loder E, Burch R, Rizzoli P (2012) The 2012 AHS/AAN guidelines for prevention of episodic migraine: a summary and comparison with other recent clinical practices guidelines. Headache 52:930–945

87. MacClellan LR, Giles W, Cole J, Wozniak M, Stern B, Mitchell BD, Kittner SJ (2007) Probable migraine with visual aura and risk of ischaemic stroke: the Stroke Prevention of Young Women Study. Stroke 38:2438–2445

88. MacGregor EA (2007) Menstrual migraine: a clinical review. J Fam Plann Reprod Health Care 33:36–47

89. MacGregor EA (2007) Migraine and use of combined hormonal contraceptives: a clinical review. J Fam Plann Reprod Health Care 33:159–169

90. MacGregor EA (2007) Migraine in pregnancy and lactation: a clinical review. J Fam Plann Reprod Health Care 33:83–93

91. MacGregor EA, Dowson A, Davies PT (2002) Mouth-dispersible aspirin in the treatment of migraine: a placebo-controlled study. Headache 42:249–255

92. MacGregor EA, Frith A, Ellis J, Aspinall L, Hackshaw A (2006) Prevention of menstruel attacks of migraine: a double blind placebo-controlled crossover study. Neurology 67:2159–2163

93. Machado RB, Pereira AP, Coelho GP, Neri L, Martin L, Luminoso D (2010) Epidemiological and clinical aspects of migraine in users of combined oral contraceptives. Contraception 81:202–208

94. Maizels M, Blumenfeld A, Burchette R (2004) A combination of riboflavin, magnesium, and feverfew for migraine prophylaxis: a randomized trial. Headache 44:885–890

95. Mannix LK, Loder E, Nett R, Mueller L, Rodgers A, Hustad CM, Ramsey KE, Skobieranda F (2007) Rizatriptan for the acute treatment of ichd-ii proposed menstrual migraine: two prospective, randomized, placebo-controlled, double-blind studies. Cephalalgia 27:414–421

96. Martin VT, Behbehani M (2006) Ovarian hormones and migraine headache: understanding mechanisms and pathogenesis - Part I. Headache 46:3–23

97. Martin VT, Behbehani M (2006) Ovarian hormones and migraine headache: understanding mechanisms and pathogenesis - Part II. Headache 46:365–386

98. Mathew NT (2003) Early intervention with almotriptan improves sustained pain-free response in acute migraine. Headache 43:1075–1079

99. Mathew NT, Schoenen J, Winner P, Muirhead N, Sikes CR (2003) Comparative efficacy of eletriptan 40 mg versus sumatriptan 100 mg. Headache 43:214–222

100. Mathew NT, Kailasam J, Meadors L (2004) Early treatment of migraine with rizatriptan: a placebo-controlled study. Headache 44:669–673

101. Mei D, Capuano A, Vollono C, Evangelista M, Ferraro D, Tonali P, Di Trapani G (2004) Topiramate in migraine prophylaxis: a randomised double-blind versus placebo study. Neurol Sci 25:245–250

102. Milla'n-Guerrero RO, Isais-Milla'n R, Benjamin TH, Tene CE (2006) N-alpha-methyl histamine safety and efficacy in migraine prophylaxis: phase III study. Can J Neurol Sci 33:195–199

103. Milla'n-Guerrero RO, Isais-Milla'n R, Barreto-Vizcaino S, Rivera-Castaño L, Garcia-Solorzano A, López-Blanca C, Membrila-Maldonado M, Muñoz-Solis R (2007) Subcutaneous histamine versus sodium valproate in migraine prophylaxis: a randomized, controlled, double blind study. Eur J Neurol 14:1079–1084

104. Milla'n-Guerrero RO, Isais-Milla'n R, Barreto-Vizcaino S, Gutiérrez I, Rivera-Castaño L, Trujillo-Hernández B, Baltazar LM (2008) Subcutaneous histamine versus topiramate in migraine prophylaxis: a double-blind study. Eur Neurol 59:237–242

105. Nappi RE, Cagnacci A, Granella F, Piccinini F, Polatti F, Facchinetti F (2001) Course of primary headaches during hormone replacement therapy. Maturitas 38:157–163

106. Newman L, Mannix LK, Landy S, Silberstein S, Lipton RB, Putnam DG, Watson C, Jöbsis M, Batenhorst A, O'Quinn S (2001) Naratriptan as short-term prophylaxis of menstrually associated migraine: a randomized, double-blind, placebo-controlled study. Headache 41:248–256

107. Nezvalova-Henriksen K, Spigset O, Nordeng H (2009) Maternal characteristics and migraine pharmacotherapy during pregnancy: cross-sectional analysis of data from a large cohort study. Cephalalgia 29:1267–1276

108. Olesen J, Diener HC, Schoenen J, Hettiarachchi J (2004) No effect of eletriptan administration during the aura phase of migraine. Eur J Neurol 11:671–677

109. Ozyalcin SN, Talu GK, Kiziltan E, Yucel B, Ertas M, Disci R (2005) The efficacy and safety of venlafaxine in the prophylaxis of migraine. Headache 45:144–152

110. Pascual J, Cabarrocas X (2002) Within-patient early versus delayed treatment of migraine attacks with almotriptan: the sooner the better. Headache 42:28–31

111. Patrick DL, Martin ML, Bushnell DM, Pesa J (2003) Measuring satisfaction with migraine treatment: expectations, importance, outcomes, and global ratings. Clin Ther 25:2920–2935

112. Pfaffenrath V, Diener HC, Fischer M, Friede M, Henneicke-von Zepelin HH (2002) The efficacy and safety of Tanacetum parthenium (feverfew) in migraine prophylaxis - a double blind, multicentre, randomized placebo controlled dose response study. Cephalalgia 22:523–532

113. Powers SW, Patton SR, Hommel KA, Hershey AD (2003) Quality of life in childhood migraines: clinical impact and comparison to other chronic illnesses. Pediatrics 112(1 Pt 1):e1–5

114. Powers SW, Patton SR, Hommel KA, Hershey AD (2004) Quality of life in paediatric migraine: characterization of age-related effects using PedsQL 4.0. Cephalalgia 24:120–127

115. Pradalier A, Auray JP, El Hasnaoui A, Alzahouri K, Dartigues JF, Duru G, Henry P, Lantéri-Minet M, Lucas C, Chazot G, Gaudin AF (2004) Economic impact of migraine and other episodic headaches in France: data from the GRIM2000 study. Pharmacoeconomics 22:985–999

116. Pradalier A, Lantéri-Minet M, Géraud G, Allain H, Lucas C, Delgado A (2004) The PROMISE study: prophylaxis of migraine with Seglor (dihydroergotamine mesilate in French primary care practice. CNS Drugs 18:1149–1163

117. Pringsheim T, Davenport WJ, Dodick D (2008) Acute treatment and prevention of menstrually related migraine headache. Neurology 70:1555–1563

118. Ravishankar K, Chakravarty A, Chowdhury D, Shukla R, Singh S (2011) Guidelines on the diagnosis and the current management of headache and related disorders. Ann Indian Acad Neurol 14(Suppl. 1):S40–59

119. Sandor PS, Di Clemente L, Coppola G, Saenger U, Fumal A, Magis D, Seidel L, Agosti RM, Schoenen J (2005) Efficacy of coenzyme Q10 in migraine prophylaxis: a randomized controlled trial. Neurology 64:713–715

120. Sandrini G, Cerbo R, Del Bene E, Ferrari A, Genco S, Grazioli I, Martelletti P, Nappi G, Pinessi L, Sarchielli P, Tamburro P, Uslenghi C, Zanchin G (2007) Efficacy of dosing and re-dosing of two oral fixed combinations of indomethacin, prochlorperazine and caffeine compared with oral sumatriptan in the acute treatment of multiple migraine attacks: a double-blind, double-dummy, randomised, parallel group, multicentre study. Int J Clin Pract 61:1256–1269

121. Saunders K, Merikangas K, Low NC, Von Korff M, Kessler RC (2008) Impact of comorbidity on headache-related disability. Neurology 70:538–547

122. Schellenberg R, Lichtenthal A, Weihling H, Graf C, Brixius K (2008) Nebivolol and metoprolol for treating migraine: an advance on beta-blocker treatment? Headache 48:118–125

123. Scher AI, Lipton RB, Stewart WF, Bigal M (2010) Pattems of medication use by chronic and episodic headache sufferersin the general population: results from the frequent headache epidemiology study. Cephalalgia 30:321–328

124. Schürks M, Rist PM, Bigal ME, Buring JE, Lipton RB, Kurth T (2009) Migraine and cardiovascular disease: systematic review and meta-analysis. Br Med J 339:b3914

125. Shaygannejad V, Janghorbani M, Ghorbani A, Ashtary F, Zakizade N, Nasr V (2006) Comparison of the effect of topiramate and sodium valproate in migraine prevention: a randomized blinded crossover study. Headache 46:642–648

126. Sheftell F, Ryan R, Pitman V (2003) Efficacy, safety, and tolerability of oral eletriptan for treatment of acute migraine: a multicenter, double-blind, placebo-controlled study conducted in the United States. Headache 43:202–213

127. Sheftell FD, Dahlof CG, Brandes JL, Agosti R, Jones MW, Barrett PS (2005) Two replicate randomized, double-blind, placebo controlled trials of the lime to onset of pain relief in the acute treatment of migraine with a fast-disintegrating/ rapid-release formulation of sumatriptan tablets. Clin Ther 27:407–417

128. Silberstein SD, Hurtchinson SL (2008) Diagnosis and treatment of the menstrual migraine patient. Headache 48:S115–123

129. Silberstein SD, Young WB, Mendizabal JE, Rothrock JF, Alam AS (2003) Acute migraine treatment with droperidol: a randomized, double-blind, placebo-controlled trial. Neurology 60:315–321

130. Silberstein S, Tepper S, Brandes J, Diamond M, Goldstein J, Winner P, Venkatraman S, Vrijens F, Malbecq W, Lines C, Visser WH, Reines S, Yuen E (2004) Randomized, placebocontrolled trial of rofecoxib in the acute treatment of migraine. Neurology 62:15520–1557

131. Silberstein SD, Elkind AH, Schreiber C, Keywood C (2004) A randomized trial of frovatriptan for the intermittent prevention of menstrual migraine. Neurology 63:261–269

132. Silberstein SD, Neto W, Schmitt J, Jacobs D, MIGR-001 Study Group (2004) Topiramate in migraine prevention: results of a large controlled trial. Arch Neurol 61:490–495

133. Silberstein SD, Freitag FG, Rozen TD, Kudrow DB, Hewitt DJ, Jordan DM, Fisher AC, Rosenthal NR (2005) Tramadol/acetaminophen for the treatment of acute migraine pain: findings of a randomized, placebo-controlled trial. Headache 45:1317–1327

134. Silberstein SD, Hulihan J, Karim MR, Wu SC, Jordan D, Karvois D, Kamin M (2006) Efficacy and tolerability of topiramate 200 mg/d in the prevention of migraine with/without aura in adults: a randomized, placebo-controlled, double-blind, 12-week pilot study. Clin Ther 28:1002–1011

135. Silberstein S, Loder E, Diamond S, Reed ML, Bigal ME, Lipton RB, AMPP Advisory Group (2007) Probable migraine in the United States: results of the American Migraine Prevalence and Prevention (AMPP) study. Cephalalgia 27:220–234

136. Silberstein SD, Saper J, Berenson F, Somogyi M, Mc-Cague MA, D'Souza JD (2008) Oxcarbazepine in migraine headache: a doubleblind, randomized, placebo-controlled study. Neurology 70:548–555

137. Silberstein SD, Holland S, Freitag F, Dodick DW, Argoff C, Ashman E (2012) Evidence-based guideline update: pharmacologic treatment for episodic migraine prevention in adults: report of the Quality Standards Subcommittee of the American Academy of Neurology and the American Headache Society. Neurology 78:1337–1345

138. Smith TR, Sunshine A, Stark SR, Littlefield DE, Spruill SE, Alexander WJ (2005) Sumatriptan and naproxen sodium for the acute treatment of migraine. Headache 45:983–991

139. Steiner TJ, Diener HC, MacGregor EA, Schoenen J, Muirheads N, Sikes CR (2003) Comparative efficacy of eletriptan and zolmitriptan in the acute treatment of migraine. Cephalalgia 23:942–952

140. Stewart W, Lipton R (2002) Need for care and perceptions of MIDAS among headache sufferers study. CNS Drugs 16(Suppl. 1):5–11

141. Stewart WF, Lipton RB, Kolodner K (2003) Migraine disability assessment (MIDAS) score: relation to headache frequency, pain intensity, and headache symptoms. Headache 43:258–265

142. Tfelt Hansen P, Pascual J, Ramadan N, Dahlof C, D'Amico D, Diener HC, Hansen JM, Lanteri Minet M, Loder E, McCrory D, Plancade S, Schwedt T, International Headache Society Clinical Trials Subcommittee (2012) Guidelines for controlled trials of drugs in migraine: Third edition. A guide for investigators. Cephalalgia 32:6–38

143. Treatment Guideline Subcommittee of the Taiwan Headache Society (2008) Treatment guidelines for preventive treatment of migraine. Acta Neurol Taiwan 17:132–148

144. Tuchman MM, Hee A, Emeribe U, Silberstein S (2008) Oral zolmitriptan in the short-term prevention of menstrual migraine: a randomized, placebo-controlled study. CNS Drugs 22:877–886

145. Vecsei L, Gallacchi G, Sagi I, Semjen J, Tajti J, Szok D, Muller M, Vadass P, Kerekgyarto M (2007) Diclofenac epolamine is effective in the treatment of acute migraine attacks. A randomized, crossover, double blind, placebo-controlled, clinical study. Cephalalgia 27:29–34

146. Vedula SS, Bero L, Scherer RW, Dickersin K (2009) Outcome reporting in industry-sponsored trials of gabapentin for off-label use. N Engl J Med 361:1963–1971

147. Winner P, Cady RK, Ruoff GE, Frishberg BM, Alexander WJ, Zhang Y, Kori SH, Lener SE (2007) Twelve-month tolerability and safety of sumatriptan-naproxen sodium for the treatment of acute migraine. Mayo Clin Proc 82:61–68

## Competing interests

MLM declares conflicts of interest with: Allergan, Almirall SAS, AstraZeneca Pharmaceuticals, GlaxoSmithKline Inc, Grunenthal, Eli Lilly & Company, Johnson & Johnson, Medtronic, Menarini, Merck, Pierre Fabre, Pfizer Inc, Sanofi-Aventis, UCB, Zambon.

DV declares conflicts of interest with: Allergan, Almirall, BMS, MSD, GSK, Janssen Cilag, Menarini, MSD, Pfizer, Sanofi-Aventis, UCB, Astra-Zeneca, Zambon.

GG declares conflicts of interest with: Allergan, AstraZeneca Pharmaceuticals, Menarini, Merck, Pfizer Inc., Zambon.

CL declares conflicts of interest with: Allergan, Almirall, AstraZeneca Pharmaceuticals, Boehringer, GlaxoSmithKline Inc, Grunenthal, Lilly, Menarini, Merck, SanofiAventis, Pfizer Inc., UCB-Schwarz-Pharma, Zambon.

AD declares conflicts of interest with: Allergan, Almirall SAS, AstraZeneca Pharmaceuticals, GlaxoSmithKline Inc, Grunenthal, Merck, Menarini, Orkyn, Pfizer Inc, Zambon.

## Authors’ contributions

MLM, DV, GG, CL and AD drafted the manuscript. All authors read and approved the final manuscript.

